# Brain water content in sudden unexpected infant death

**DOI:** 10.1007/s12024-023-00584-8

**Published:** 2023-02-03

**Authors:** Johanna Marie Lundesgaard Eidahl, Torleiv Ole Rognum, Arne Stray-Pedersen, Siri Hauge Opdal

**Affiliations:** 1 Division of Laboratory Medicine, Department of Forensic Sciences, Oslo University Hospital, Oslo, Norway; 2 Institute of Clinical Medicine, University of Oslo, Oslo, Norway

**Keywords:** Aquaporin-4, Brain edema, Brain water content, SIDS, Sudden infant death syndrome

## Abstract

The extensive and rapid development of the human brain during the first years of life complicates the postmortem diagnosis of brain edema in infancy. The aim of this study was to describe brain water content, the brain weight/body weight ratio, and the brain weight/head circumference ratio throughout the first years of life. Furthermore, we examined the relationship between these parameters and rs2075575 in the AQP4 gene. Our hypothesis was that dysregulated water homeostasis might be a risk factor for sudden infant death syndrome (SIDS), which may be reflected by increased water content in the brain. The study included 90 subjects with sudden unexpected death < 4 years of age: 22 cases of sudden infant death syndrome, 11 cases of sudden unexplained death in childhood, 47 cases of death due to disease, and 10 cases of accident/violent death. Brain water content, brain weight/body weight ratio, and brain weight/head circumference ratio were investigated according to corrected age, diagnosis group, attempt to resuscitate, and presence of brain edema. We found that brain water content and brain weight/body weight ratio were significantly reduced with increasing age, while brain weight/head circumference were increased. Brain weight/head circumference was correlated with brain water content. Cases with brain edema had a significantly higher brain weight/head circumference than the non-edematous cases. No differences were found between the diagnosis groups for any of the investigated parameters. In summary, the findings contribute to the current body of knowledge regarding brain growth during the first months of life.

**Supplementary Information:**

The online version contains supplementary material available at 10.1007/s12024-023-00584-8.

## Introduction

The brain undergoes rapid and complex development during the first months of life, particularly with respect to synaptic development and homeostatic control. This cerebral maturation also includes a significant increase in lipid concentration due to myelination [1]. Together, these extensive cerebral changes make postmortem evaluation of brain edema in children and infants difficult.

Sudden infant death syndrome (SIDS) is defined as the sudden unexpected death of an infant < 1 year of age, with the onset of the fatal episode apparently occurring during sleep that remains unexplained after a thorough investigation, which includes the performance of a complete autopsy and a review of the circumstances of death and clinical history [2]. The definition of sudden unexpected death in childhood (SUDC) is similar, except it comprises children older than 1 year of age [3]. Several studies have indicated that vulnerabilities in the development and regulation of brain function are involved in SIDS, including both delayed myelination and vagal nerve maturation [4–8]. In addition, increased brain weight has been reported in SIDS, and despite conflicting data, this has been suggested to reflect abnormal cerebral development that might be detrimental to vital neural control [9–13].

Aquaporin 4 (AQP4), the main water channel in the brain, is responsible for water homeostasis and neural signal transduction [14, 15]. In humans, AQP4 is present from an early gestational age, with increasing vascular coverage throughout pregnancy. This may indicate a close relationship between water transport regulation and brain development [16].

The gene encoding AQP4 is located on the long arm of chromosome 18, and there are reports of variants giving both reduced and increased water permeability [17, 18]. Single nucleotide polymorphisms (SNPs) in the AQP4 gene are associated with both seizure susceptibility, epilepsy, and the development of brain edema [19, 20]. It is also suggested that AQP4 gene variation may be involved in the development of hematoma and edema formation after intracerebral hemorrhage [21].

A previous study of the AQP4 gene in Norwegian subjects with SIDS indicated an association between the age at death, the brain weight/body weight ratio, and AQP4 rs2075575 genotypes [22]. The CT/TT genotypes were more frequent in SIDS subjects than in controls, and the same genotypes were associated with a high brain weight/body weight ratio in SIDS subjects who were less than 12 weeks of age. Rs2075575 is a tag SNP located in the promoter region of the AQP4 gene, and it is thus unlikely that this SNP alters the function of the AQP4 protein. However, one might speculate that the SNP influences the expression of the gene.

The aim of this study was to describe brain water content, the brain weight/body weight ratio, and the brain weight/head circumference ratio throughout the first years of life. Furthermore, we examined the relationship between these parameters and rs2075575 in the AQP4 gene. We hypothesize that dysregulated water homeostasis may be a risk factor for SIDS, which may be reflected by increased water content in the brain.

## Materials and methods

### Individuals

Subjects were prospectively collected from autopsies performed at the Department of Forensic Sciences, Oslo University Hospital during the period 2010–2017. The inclusion criteria were infants and children with sudden unexpected deaths who were < 4 years of age and had a postmortem time of less than 48 h. Subjects with severe brain injury or mutilation were excluded. In total, 90 infants and young children (45 males and 45 females) with sudden unexpected deaths were included in the study. Of these, there were 22 subjects with SIDS, 11 subjects with SUDC, 47 subjects with death due to disease, and 10 subjects with accidental/violent death (Table 1).

**Table 1:** Survey of the subjects included in this study. All parameters are given as medians and ranges. The specific diagnoses in the disease group are given in Online Resource 1

	22 SIDS < 1 year of age	11 SUDC > 1 year of age	47 disease	10 violent death
Cause of death	15 SIDS I 7 SIDS II/ undetermined	8 SUDC 3 undetermined	15 infection 14 neonatal 18 others	6 suffocation 2 drowning 2 traumatic
Sex (male/female)	14/8	5/6	20/27	6/4
Age, weeks	12.9 (0.9–41.3)	72.4 (53.7–117.9)	8.0 (0–161.6)	73.4 (1.9–180.4)
Corrected age, weeks	10.5 (-7–41)	72 (54–118)	7.8 (-6–161)	73.5 (0–180)
Postmortem time, hours	23 (7–87)	15 (2–41)	26 (3–144)	25.1 (12–42)

All SIDS/SUDC subjects were classified according to the San Diego definition and the SUDC definition proposed by Krous et al. [2, 3], applying the criteria used in the Nordic SIDS study [23]. The investigation protocol included an evaluation of the circumstances of death, a review of the medical and family history, a total skeletal radiography examination, and a thorough autopsy with extensive histologic and microbiologic examinations and genetic testing for long QT syndrome and medium-chain acyl-CoA dehydrogenase deficiency. Of the 33 subjects with SIDS/SUDC, 23 were classified as having pure SIDS/SUDC, corresponding to category I SIDS according to the San Diego definition and meeting the requirements for being unexplainable in the new classification recommendations [24]. Another 10 subjects (7 in the SIDS group and 3 in the SUDC group) were classified as having category II SIDS using the San Diego definition, corresponding to undetermined in the new classification recommendations [24]. All subjects were from the southeastern part of Norway, and 70% of the subjects were of Caucasian ethnicity.

Information regarding age, sex, brain and body weight, external head circumference, the presence of brain edema, the cause of death, and the circumstances of death (including information regarding attempts to resuscitate) were registered during the postmortem examination. Age was corrected according to gestational age at birth. Corrected age was used for all comparisons in the current paper.

### Evaluation of brain edema

A macroscopic examination for brain edema was performed during the autopsy immediately following the removal from the skull. Features such as bulging and gyral flattening, impression marks on the base of the brain, and compression of the ventricles were assessed. The brains were subsequently submerged in 10% formalin for fixation. After fixation, the macroscopic edema evaluation was verified by a neuropathologist trained and experienced in forensic cases, before sampling and microscopic examination by the same neuropathologist. Only subjects with clear gyral flattening and compression of the lateral ventricles were designated as having edema. In dubious cases, the microscopic confirmation of key reactions, such as red neurons suggestive of hypoxic-ischemic injury in conjunction with pale and sieve-like myelin and vacuolar appearance of grey matter neuropil, were interpreted as true edema. Ambiguous or no histological signs of edema or vital reactions to hypoxia were regarded as postmortem congestion and classified as “no edema.” The subjects were thus categorically classified as having either “edema” or “no edema” based on the overall results of these evaluations.

### Determination of brain water content

All sampling was performed during the autopsy. Small cubic samples of brain tissue approximately 1 cm^3^ in size were excised from the surface of the right frontal, left and right temporal, and right occipital lobes, as well as from the posterior right portion of the cerebellum. The samples, consisting of approximately equal amounts of gray and white matter, were taken within a few minutes after removing the brain from the skull, immediately after performing the macroscopic examination and weight measurement, but prior to formalin fixation. Areas usually sampled for microscopic examination were avoided to enable further tissue sampling for diagnostic purposes. The research tissue samples were kept in small plastic containers with airtight lids. After calibration of the containers, the wet weight of the samples was measured using a precision scale in grams with four decimal places. Thereafter, the containers with samples were stored at − 76 °C. Following this, the water content of the samples was determined by drying them for 24 h at 95 °C in an oven attached to a vacuum pump. The remaining solid material weight was then assessed for each sample. The result was expressed as the brain water content percentage: (wet weight – dry weight)/(wet weight) × 100% [25].

### Brain weight/body weight ratio

Body weight and fresh brain weight were registered during the autopsy. The ratio between brain weight and body weight was calculated for each subject and expressed as a percent (brain weight/body weight × 100%).

### Brain weight-head circumference ratio

External head circumference was measured by a standard procedure using a measuring tape around the widest part of the skull. The ratio between the brain weight and head circumference was calculated for each subject (brain weight/head circumference).

### Genotyping

DNA was extracted from the spleen using the QiAmp DNA Minikit and the BioRobotEZ (Qiagen, Hombrechtikon, Switzerland). The SNP rs2075575 was genotyped using TaqMan SNP genotyping assay (Applied Biosystems, Foster City, CA) and an ABI 7500 real-time PCR machine (Thermo Fisher Scientific, Waltham, MA, USA) according to the manufacturer’s instructions.

### Statistical analysis

Average water content was analyzed according to corrected age, diagnosis, the brain weight/body weight ratio, the brain weight/head circumference ratio, the presence of brain edema, and attempts to resuscitate using the Mann–Whitney U test when comparing two groups and the Kruskal–Wallis test for more than two groups. The chi-square test was used to evaluate attempts to resuscitate according to brain edema and rs2075575 genotype according to the corrected age group. All statistical analyses were performed using IBM SPSS Statistics, version 25.0 (SPSS, Chicago, IL, USA).

## Results

**Fig. 1:**
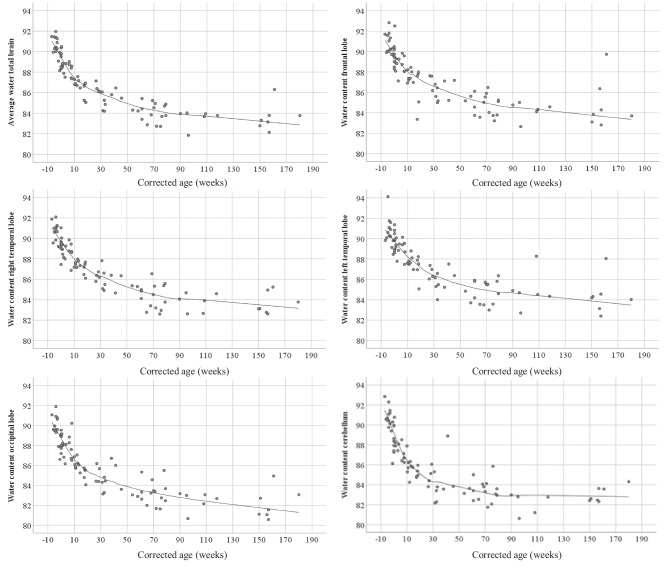
Brain water content in each of the regions (frontal lobe, right and left temporal lobe, occipital lobe, and cerebellum), in addition to the average water content. The plots include both subjects with edema and subjects without edema

The brain water content in each of the sampled regions as well as the average water content is given in Table 2 and Fig. 1.

**Table 2:** Summary of results according to diagnosis groups. All parameters are given as medians and ranges

Diagnosis group	22 SIDS < 1 year of age	11 SUDC > 1 year	47 disease	10 violent death
Brain weight/body weight ratio (%)	11.8 (9–16.5)	11.5 (6.5–12.5)	11.6 (6.4–17.3)	9.6 (7.1–13.1)
Brain weight/head circumference ratio	16.1 (9.6–22.5)	24.5 (21.9–27.4)^c^	13.7 (8.4–28.4)^d^	25.8 (14.4–27.5)
Average water content (%)	87.35 (84.20–91.46)	83.78 (81.85–84.54)	87.36 (82.78–91.96)	84.97 (82.14–88.41)
Right frontal lobe (%)	87.80 (84.99–95.64)^a^	84.56 (82.65–85.90)	88.53 (83.09–92.82)^e^	84.94 (82.80–88.30)
Right temporal lobe (%)	87.30 (84.65–91.88)^b^	84.11 (82.59–85.36)	87.97 (82.66–92.08)^e^	85.15 (82.61–88.98)
Left temporal lobe (%)	87.90 (84.01–91.37)	84.34 (82.70–85.91)	88.27 (83.13–94.12)^d^	85.75 (82.39–88.80)
Right occipital lobe (%)	86.23 (83.13–91.06)^a^	82.62 (80.67–83.47)	86.60 (81.07–91.90)	84.95 (80.57–88.01)
Right cerebellum (%)	86.56 (82.17–92.84)	82.77 (80.62–83.41)	86.00 (81.21–92.29)^e^	84.31 (82.83–87.94)^c^

^a^ *n* = 21

^b^ *n* = 20

^c^ *n* = 9

^d^ *n* = 45

^e^ *n* = 46

### Brain water content according to corrected age

**Fig. 2:**
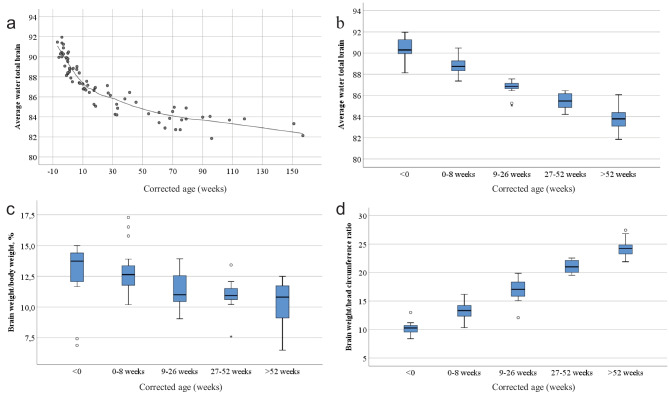
Brain water content according to corrected age in subjects without brain edema. **a** Average water content according to corrected age. **b** Average water content according to the corrected age group. **c** Brain weight/body weight ratio according to corrected age group. **d** Brain weight/head circumference according to the corrected age group. For plots b-d, each box contains the middle 50% of the values, the thick lines indicate the median, and the bars indicate the maximum and minimum values

We observed that the average brain water content in subjects categorized as not having edema (*n* = 75) was highest for newborns and declined steadily with increasing age (*p* < 0.001) (Fig. 2a, b). This trend was noted in all parts of the brain (Fig. 1) and did not differ when including subjects with brain edema. The same pattern was found for the brain weight/body weight ratio, with the ratio being highest at birth and then declining with increasing age (*p* < 0.001) (Fig. 2c). Additional data regarding brain weight and body weight can be found in Online Resources 3 and 4, respectively.

For the brain weight/head circumference ratio, the highest ratio was found in the oldest infants (*p* < 0.001) (Fig. 2d). The brain water content in each of the sampled regions as well as the average water content, brain weight/body weight, and brain weight/head circumference ratio according to corrected age are given in Online Resource 2.

**Fig. 3:**
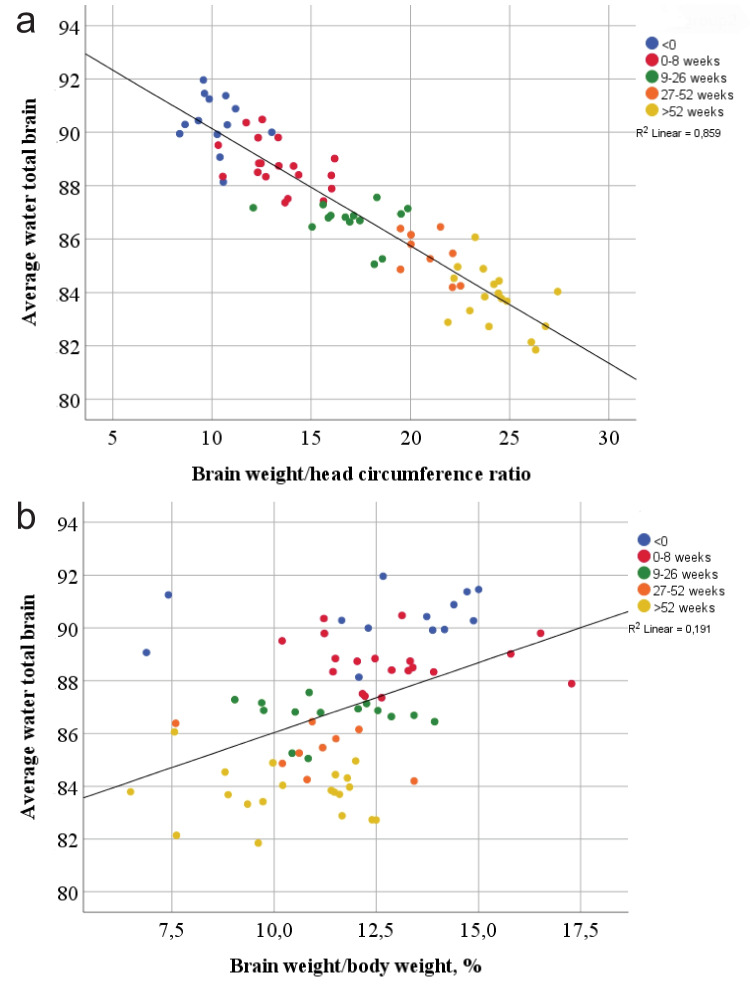
**a** Average brain water content relative to the brain weight/head circumference ratio. **b** Average water content relative to brain weight/body weight. The plots include only subjects without brain edema

Furthermore, a significant correlation was found between average brain water content and the brain weight/head circumference ratio; the infants with the highest average brain water content also had the lowest brain weight/head circumference ratio (R^2^ linear = 0.859, *p* < 0.001) (Fig. 3a). This corresponded to the smallest infants having the smallest brain weight/head circumference ratio, as illustrated above. No correlation between the brain weight/body weight ratio and average brain water content was found (*R*^2^ = 0.191) (Fig. 3b).

### Brain water content according to diagnosis

When comparing brain water content between SIDS/SUDC subjects and controls, corrected age must be taken into consideration. With regard to SIDS, it was possible to compare 13 SIDS with 13 disease controls when allowing a maximum age difference of 1 week. However, this did not result in any difference in average brain water content (*p* = 0.55). For the SUDC subjects (> 52 weeks of corrected age), it was possible to compare 11 SUDC with 13 cases of disease and 7 cases of violent death. For this group, there were no differences between the cases and controls (*p* = 0.36 and *p* = 0.21, respectively).

### Brain water content according to the presence of brain edema, corrected age group > 52 weeks

**Fig. 4:**
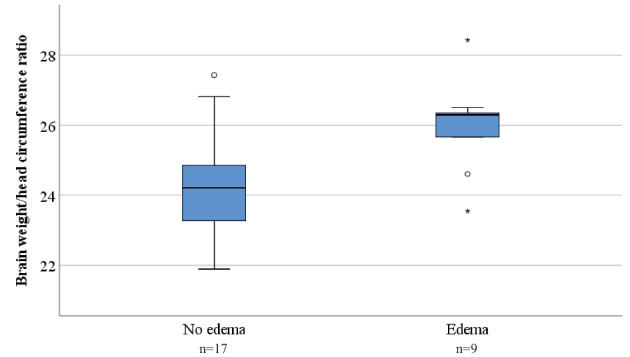
Brain weight/head circumference ratio according to the presence of brain edema in the age group > 52 weeks. Each box contains the middle 50% of the values, the thick line indicates the median, and the bars indicate the maximum and minimum values

In total, 15 of the subjects (16.7%) had verified brain edema (1 SIDS, 7 diseases, and 7 violent deaths) (Online Resource 2). Of these, 10 subjects were in the age group > 52 weeks (4 diseases and 6 violent deaths). In the same age group, 20 subjects did not have brain edema. Information regarding head circumference was available for 26 of the subjects. Subjects with brain edema (*n* = 9) had a significantly greater brain weight/head circumference ratio than subjects without edema (*n* = 17) (*p* = 0.025) (Fig. 4). No differences in the age group > 52 weeks were found for the brain weight/body weight ratio or total brain water content (*p* = 0.27 and *p* = 0.19, respectively). It was not possible to perform comparisons in any of the other age groups due to a low number of subjects with brain edema.

### Brain water content according to resuscitation, corrected age group > 52 weeks

**Fig. 5:**
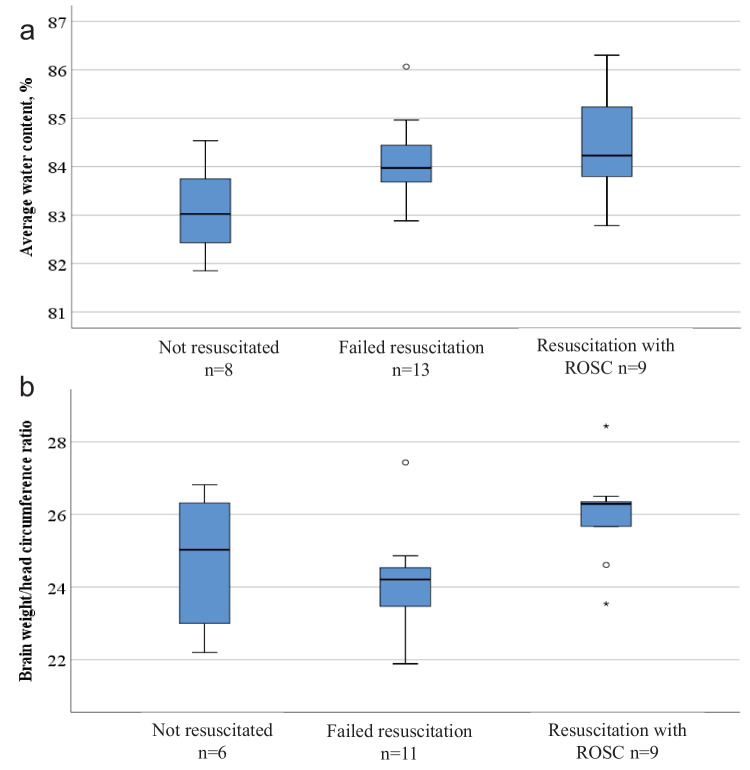
Variables according to resuscitation attempts in the subjects aged > 52 weeks. **a** Average water brain water content according to resuscitation attempts. **b** Brain weight/head circumference according to resuscitation attempts. Each box contains the middle 50% of the values, the thick line indicates the median, and the bars indicate the maximum and minimum values

Resuscitation was attempted for 79 of the subjects, and for 16 subjects, the attempts led to the transient return of spontaneous circulation (ROSC), but death occurred within 72 h after the initial collapse. Edema was observed in 14 out of these 16 cases, all with signs of hypoxic-ischemic brain injury. However, due to the small number of subjects, calculations regarding attempts to resuscitate were only performed in the corrected age group > 52 weeks. There was a significant difference in brain water content among subjects with no resuscitation attempts, subjects who had undergone resuscitation attempt but with no ROSC, and subjects with initial successful resuscitation treated for 3–72 h before the final stop in circulation (*p* = 0.019) (Fig. 5a). The average water content was higher in both subjects with failed resuscitation attempts and subjects with ROSC compared to subjects for whom resuscitation was not attempted (*p* = 0.027 and *p* = 0.007, respectively) (Fig. 5a). For the brain weight/head circumference ratio, there was a significant difference only between subjects with failed resuscitation attempts hours and those with ROSC (*p* = 0.061 for all three groups, *p* = 0.010 for failed resuscitation vs. initial ROSC) (Fig. 5b). No differences in the brain weight/body weight ratio were found with regard to resuscitation attempts (data not shown). All the subjects in the age group > 52 weeks with resuscitation attempts with ROSC (*n* = 9) had verified brain edema (*p* < 0.001).

### Brain water content according to genotype

The genotyping of rs2075575 in the AQP4 gene was available for 87 of the subjects. When investigating water content according to genotype, a significant difference between the genotypes CC vs. CT/TT was found (*p* = 0.05). However, we discovered that this was due to a skewed age distribution of the material: 72% of the subjects with a CC genotype were < 8 weeks of age, while 68% of the subjects with a T allele were > 8 weeks of age (*p* = 0.02) (Table 3). Thus, when taking age into account, no correlations were found between the rs2075575 genotype and average brain water content.

**Table 3:** Number of subjects and genotype distribution of rs2075575 in the AQP4 gene according to age group

Corrected age	≤ 8 weeks	> 8 weeks
CC	13 (72%)	5 (28%)
CT/TT	22 (32%)	47 (68%)

## Discussion

The main finding of this study is the significant difference in water content in the brains of infants and toddlers according to age. The average brain water content was highest in the youngest children (corrected age group < 0) and then declined during the first year of life as the brain developed (Figs. 1 and 2a, b). Simultaneously, it is interesting that AQP4 expression in the brain also seemed to be age-dependent [26]. The expression of AQP4-positive astrocytes in the hippocampus was found to be highest in infants ≤ 12 weeks of age, decreasing in the group between 12 and 52 weeks of age, and lowest in the group > 52 weeks of age [26]. Furthermore, our study confirms the findings of Dobbing and Sands in the study of human brains ranging in age from 10 weeks gestational age to 7 postnatal years [27]. They found that the water content was highest around birth and then declined during the first year of life. The study also found that the process of increasing lipid content in the brain was related to the rapidly declining water content. This may be of particular interest, as myelination of neuronal axons is an important aspect of postnatal brain development [27].

An edematous brain contains more water than a non-edematous brain. Even so, we were unable to establish an increased water content in the tissue samples from the edematous cases. This may be due to a slightly uneven distribution of edema fluid, despite the edema being global. If most of the edema fluid is retained in the white matter, as indicated in several studies [28–30], our sampling method would be less optimal, as our samples only contained 50% (~ 0.5 g) white matter.

The brain weight/body weight ratio is commonly used when evaluating brain development in children and infants, and the present study demonstrates that the ratio declines as infants get older (Fig. 2c, Online Resources 3, 4, and 5). However, the range within each age group is wide, underlining the idea that the brain weight/body weight ratio is only useful for rough evaluations.

An increased brain weight/head circumference ratio significantly correlates with increasing age (Fig. 2d). The idea behind this ratio originated from the need for more objective measurements for enlarged brains in general and brain edema in particular [25]. The ratio originally included the inner skull circumference measured in an adult population using CT scanning [25]. In children and infants, a more common practice is to measure the head circumference as a part of the external examination of the body, making the brain weight/head circumference ratio easily accessed in the autopsy room. We suggest that the brain weight/head circumference ratio may be a better measure for brain size and development than the brain weight/body weight ratio, given the smaller overlap between each age group (Fig. 2c, d). This was further strengthened by the strong correlation between this ratio and average brain water content (*R*^2^ = 0.859) (Fig. 3a), and the lack of correlation between the brain weight/body weight ratio and average brain water content (*R*^2^ = 0.191) (Fig. 3b).

With regard to average brain water content in the presence of brain edema, the only age group containing enough subjects was the age group > 52 weeks (Fig. 4). In this group, 10 subjects had verified brain edema, of which 9 had available measurements of head circumference. Subjects with brain edema had a significantly higher brain weight/head circumference ratio than subjects without brain edema (Fig. 4, *p* = 0.025). The same level of significance was not found for brain weight/body weight or for average brain water content, indicating that the brain weight/head circumference ratio may also be used for evaluating the presence of brain edema in children and small infants.

Most of the cases with verified edema had been resuscitated and spontaneous circulation was temporarily established. We interpreted the edema to be a feature of hypoxic-ischemic cerebral injury leading to neuronal cell death and water accumulation in the brain tissue (Fig. 5a). Subjects with attempts of resuscitation, but no ROSC also had significantly increased water content. This may reflect a process with early-phase hypoxic injury. There was no significant difference with regard to brain weight/head circumference ratio between resuscitated cases and non-resuscitated cases (Fig. 5b). The sample size available for analysis was small as only cases > 52 weeks of age could be compared due to the changes in maturation occurring in infancy.

Several studies have rejected the hypothesis of enlarged brains in SIDS subjects due to a lack of significant findings when the brain weight/body weight ratio is explored [9, 31–33]. The results of the present study also indicate that the method using the brain weight/body weight ratio lacks the precision needed to detect differences. Furthermore, the selection of controls will highly influence the results, and age-matched controls without disease are not easily obtained. Thus, the role of brain weight in SIDS and whether it is enlarged remains controversial.

AQP4 is the main water channel in the brain and is thus involved in the development of brain edema [34]. Both gain-of-function and loss-of-function gene variants are present in the AQP4 gene, including an association between rs9951307 and severe brain edema in patients with middle cerebral artery occlusion [17, 18, 20]. Part of the aim of the present study was to investigate the relationship between rs2075575 in the AQP4 gene and brain water content. The CT/TT genotypes of this SNP have previously been reported to be associated with SIDS [22]. However, in our population, the apparent association between a C allele in this SNP and water content was due to a skewed age distribution in the material rather than genotype.

This study was subject to a few limitations. The presence of brain edema was classified by macroscopic evaluation, first by the forensic pathologists at the time of the autopsy, then verified by a neuropathologist after the brain was formalin-fixed. This evaluation is subjective. Postmortem CT imaging and photography would provide means for secondary assessment; however, this information was only available in the cases autopsied from 2014 and forward, which is about half of the cases. The mode of resuscitation differed from subject to subject, from cardiopulmonary resuscitation only, to resuscitation involving various medical treatments, including the administration of fluids. Furthermore, due to the limited sample size and when taking age into account in particular, the data should be considered preliminary. Alas, age-matched non-disease controls are not easily obtained.

In conclusion, the findings in the present study may provide increased knowledge regarding brain growth during the first months of life. A significant reduction was observed in brain water content as the brain developed during the first year of life. The brain weight/body weight ratio was significantly reduced, while the brain weight/head circumference ratio increased. We suggest that the brain weight/head circumference ratio represents a better measure for brain enlargement, as the range within each corrected age group is narrower than that of the brain weight/body weight ratio.

## Key points

1. Brain water content and brain weight/body weight ratio were highest at birth and decreased with increasing age.
2. The brain weight/head circumference increased with increasing age.
3. Subjects with brain edema had significantly increased brain weight/head circumference ratio, but no differences in brain water content using samples from the brain surface.
4. There were no differences in brain water content between SIDS cases and controls.

### Supplementary Information

Below is the link to the electronic supplementary material.

Online Resource 1 (PDF 104 KB)

Online Resource 2 (PDF 15 KB)

Online Resource 3 (PDF 240 KB)

Online Resource 4 (PDF 241 KB)

Online Resource 5 (PDF 213 KB)

## Data Availability

The dataset generated and analyzed during the current study is available from the corresponding author upon reasonable request.

## Abbreviations

AQP4: Aquaporin 4
ROSC: Return of spontaneous circulation
SIDS: Sudden infant death syndrome
SUDC: Sudden unexplained death in childhood
