# Supplementary material for: Brain water content in sudden unexpected infant death
Source: Forensic Sci Med Pathol. 2023 Feb 3;19(4):507–16. doi: 10.1007/s12024-023-00584-8 (PMC10752850; doi:10.1007/s12024-023-00584-8)
Supplement: Supplementary file 1 — Online Resource 1 (PDF 104 KB) [file 12024_2023_584_MOESM1_ESM.pdf]

Brain water content in sudden unexpected infant death
Forensic Science, Medicine and Pathology

Online Resource 1

Cause of death in the 47 cases of disease. Age is given as median and range.

**Disease (n=47)**	**Sex (m/f)**	**Age (weeks)**	**Corrected age (weeks)**	**Specifics**
**Infection (n=15)**	8/7	8 (0.1-161.6)	7.6 (-4-161)	7 septicemia<br>5 pneumonia<br>1 cytomegalovirus infection<br>1 myocarditis<br>1 meningitis
**Neonatal (n=14)**	6/8	0 (0-0.3)	-1.5 (-6-2)	4 death due to hypoxia during birth<br>3 placenta failure or –pathology<br>6 cardiac, pulmonary or circulatory failure<br>1 anion infection syndrome
**Others (n=18)**	6/12	38.6 (1.9-155.9)	31 (0-156)	10 heart- or circulatory failure after disease<br>2 intestinal disease<br>1 nemaline myopathy<br>1 MCAD-deficiency<br>1 rhabdomyolysis<br>1 Pierre Robins syndrome<br>1 heart failure after severe dehydration<br>1 brain tumor
