# Supplementary material for: Brain water content in sudden unexpected infant death
Source: Forensic Sci Med Pathol. 2023 Feb 3;19(4):507–16. doi: 10.1007/s12024-023-00584-8 (PMC10752850; doi:10.1007/s12024-023-00584-8)
Supplement: Supplementary file 2 — Online Resource 2 (PDF 15 KB) [file 12024_2023_584_MOESM2_ESM.pdf]

Brain water content in sudden unexpected infant death
Forensic Science, Medicine and Pathology

Online Resource 2

Brain water content according to corrected age. Each parameter is given as median and range.

**Corrected age group**	**<0 weeks**	**0-8 weeks**	**9-26 weeks**	**27-52 weeks**	**>52 weeks**
**Number of cases**	14	21	14	11	30
**Cases with edema**	1	2	0	2	10
**Brain weight / body weight ratio**	13.2 (6.9-15)	12.6 (6.4-17.3)	11 (9-13.9)	10.8 (7.6-13.4)	10.1 (6.5-13.1)
**Brain weight / head circumference ratio**	10.16 (8.4-13)	13.3 (9.6-16.1)	17.05 (12.1-19.9)	21 (19.5-22.5)	24.6 (21.9-28.4)[c]
**Avg water content, %**	90.36 (88.14-91.46)	88.58 (87.36-91.48)	86.84 (85.06-87.56)	85.80 (84.2-86.45)	83.82 (81.85-86.30)
**Right frontal, %**	90.35 (89.65-92.82)[a]	89.14 (87.11-92.49)[b]	87.48 (83.35-95.64)	86.44 (84.99-87.61)	84.57 (82.65-89.93)
**Right temporal, %**	90.90 (89.16-92.08)[a]	89.09 (86.86-91.03)[b]	87.32 (85.79-87.97)[a]	86.33 (84.65-87.82)	84.08 (82.59-87.07)
**Left temporal, %**	90.44 (88.45-94.12)	89.39 (87.46-91.37)[b]	87.57 (85.07-88.79)	86.28 (84.01-87.50)	84.56 (82.39-88.27)[d]
**Right occipital, %**	89.60 (86.60-91.90)[a]	88.11 (86.17-90.22)	85.72 (84.06-87.05)	84.42 (83.13-86.71)	82.95 (80.57-85.50)
**Right cerebellar, %**	90.52 (86.13-92.84)	88.02 (86.24-90.78)[b]	85.54 (83.81-87.90)	83.84 (82.17-88.89)	83.08 (80.62-85.86)[d]

a: n=13 b: n=20 c: n=26 d: n=29
