# Supplementary material for: Brain water content in sudden unexpected infant death
Source: Forensic Sci Med Pathol. 2023 Feb 3;19(4):507–16. doi: 10.1007/s12024-023-00584-8 (PMC10752850; doi:10.1007/s12024-023-00584-8)

Brain water content in sudden unexpected infant death
Forensic Science, Medicine and Pathology

Online Resource 5

Head circumference according to corrected age.

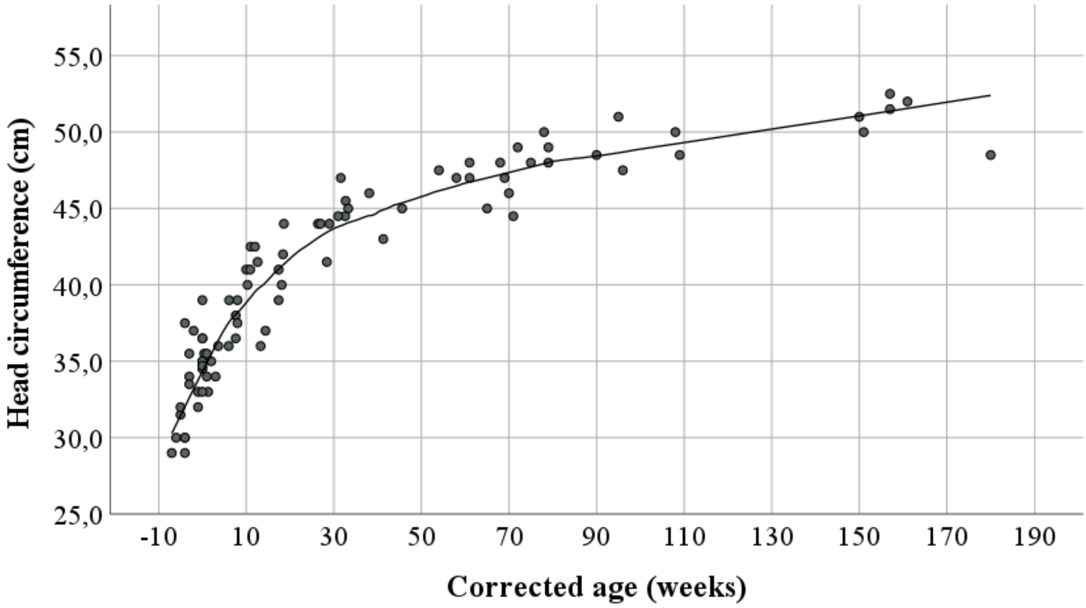

Supplement: Supplementary file 5 — Online Resource 5 (PDF 213 KB) [file 12024_2023_584_MOESM5_ESM.pdf]
